# Impact of TBT on the vitellogenesis and sex hormones in freshwater prawn *Macrobrachium rosenbergii* (De Man, 1879)

**DOI:** 10.1186/2046-9063-9-10

**Published:** 2013-05-01

**Authors:** Peranandam Revathi, Palanisamy Iyapparaj, Lourduraj Arockia Vasanthi, Natesan Munuswamy, Muthukalingan Krishnan

**Affiliations:** 1 Department of Environmental Biotechnology, Bharathidasan University, Trichy 620 024, Tamil nadu, India; 2 CAS in Marine Biology, Faculty of Marine Sciences, Annamalai University, Parangipettai 608 502, Tamil nadu, India; 3 Department of Zoology, University of Madras, Guindy Campus, Chennai 600 025, Tamil nadu, India

**Keywords:** *Macrobrachium rosenbergii*, Tributyltin, Ovarian development, Vitellogenesis, Sex hormones

## Abstract

**Background:**

Tributyltin (TBT) is a ubiquitous persistent xenobiotic that can be found in freshwater, estuarine and marine ecosystem. TBT is a strong endocrine disrupting compound (EDC) that can cause toxic threat to aquatic organisms. Imposex, sexual deformities and endocrine dysfunctions are the causes of TBT to most of the aquatic organisms. Effect of TBT on the vitellogenesis and sex hormonal changes in *Macrobrachium rosenbergii* has never been reported. Hence, the present investigation was undertaken to find out the impact of TBT on histological changes in the different reproductive tissues, sex hormonal alterations and level of biomarkers like vitellogenin and vitellin in *M. rosenbergii*.

**Results:**

The present investigation documents the possible impact of tributyltin (TBT) on the vitellogenesis in freshwater female prawn *M. rosenbergii*. TBT at 10 ng/l, 100 ng/l and 1000 ng/l concentrations were exposed individually to prawns for a period of three months. At higher concentration of 1000 ng/l, the ovarian development was arrested and ovary remained at spent stage. At lower concentration of TBT (10 ng/l), the development proceeded up to early vitellogenic stage. At intermediate concentration of 100 ng/l TBT, the ovary remained at pre vitellogenic stage and thereafter no development was noticed. Histological results indicated the normal ovarian development with vitellogenic oocytes, filled with yolk globules in control prawn. On the other hand, the TBT treated groups showed reduction in yolk globules, fusion of developing oocytes and abundance of immature oocytes. Immunofluorescence staining denoted the remarkable reduction in vitellin content in ovary of TBT treated prawn. Hence, TBT had conspicuously inhibited the vitellogenesis by causing hormonal imbalance in *M. rosenbergii*.

**Conclusion:**

TBT had notably inhibited the vitellogenesis due to hormonal imbalance. This endocrine dysfunction ultimately impaired the oogenesis in the freshwater female prawn *M. rosenbergii*.

## Background

Freshwater ecosystem is under increasing threat due to rapid expanding population and the subsequent modernization process resulted in invisible exploitation of natural resources leading to pollution. Rivers are very vulnerable towards pollution, since the industrial, domestic and farm effluents are directly released into them. During the past few decades, rising trends of population explosion, development of modern technology, industrialization and dramatic increase in the production and consumption of large variety of new synthetic chemicals were reported. These kinds of modernization are the reason behind the enormous release of pollutants into aquatic environment [1]. Accumulation of industrial effluents and agricultural runoff in water bodies has become a major concern [2]. TBT is an ubiquitous persistent xenobiotic that can be found in freshwater, estuarine and marine ecosystem [3].

Organotin compounds, particularly TBT, have been reported to be strong endocrine disrupting compound (EDC). TBT is highly toxic to many aquatic organisms and is still detected in aquatic environments though it had banned in antifouling paints because of its usage as biocides in a variety of consumer and industrial products. The level of TBT in the aquatic environment is still cause of great concern [4]. Effects of TBT have been investigated in several aquatic organisms, including algae [5] and crustaceans [6]. Champ [7] reported that TBT inhibits growth, reproduction [8,9] and sexual differentiation in fishes [10]. In our previous study, we documented the inhibition of organogenesis as well as embryonic development in *M. rosenbergii* due to TBT toxicity [11].

Histological analysis appears to be a very sensitive parameter and is crucial in determining cellular changes that may occur in target organs [12]. Besides, biochemical parameters are the best indicators of stress situations caused by xenobiotics [1]. Vitellogenesis is a biomarker of reproductive disruption by xenobiotics [13]. As evidence, a wide range of agrochemicals, industrial and municipal contaminations can decrease gonadal development and steroid levels [14]. Sex hormones derived from the gonads play crucial roles in sexual differentiation, maturation and behavior in vertebrates [15]. It is well known that 17β-estradiol once secreted into the circulation, stimulates the hepatic production of vitellogenin, necessary for oocyte maturation [16]. Some studies have additionally assessed the impact of xenobiotics on endogenous steroid levels, which may in turn be an indication of altered steroid synthesis and metabolism [17].

Extensive literatures dealing with the adverse impact of TBT in molluscs but in contrast only a few articles have addressed the effects of TBT on crustaceans. With reference to TBT toxicity, not much information is available on freshwater organisms especially on the commercially important species *M. rosenbergii*[11]. Therefore, the present study was conducted to examine the impact of TBT on vitellogenesis and sex hormones in freshwater female prawn, *Macrobrachium rosenbergii*.

## Results

In the present study the impact of TBT on vitellogenesis in the adult freshwater female prawn *M. rosenbergii* was studied with the analysis of survival rate, growth as a measure of body weight, GSI, HSI, histological, immunofluorescence, biochemical changes and quantification of vitellogenin, vitellin content and female specific hormones in different reproductive tissues of both control and TBT treated prawns.

### Survival rate and bodyweight

**Figure 1:**
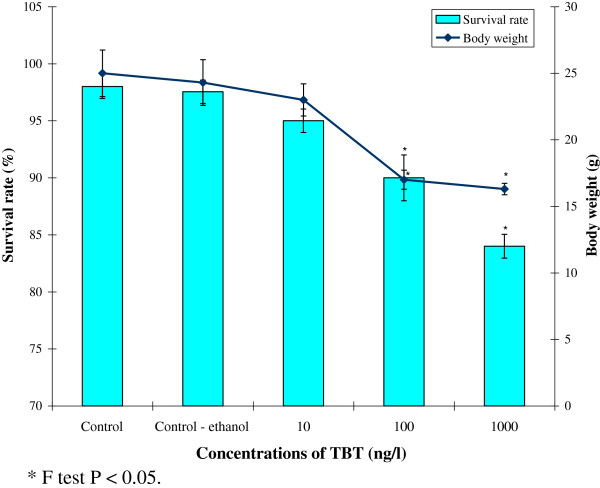
Impact of TBT on the survival rate and body weight of M. rosenbergii. * F test P < 0.05.

The maximum survival rate of 98.0 ± 1.05% was obtained in the control group. However, in the TBT treated groups, survival rate was decreased compared to control group. At 10 ng/L, 100 ng/Land 1000 ng/L, the survival rate was decreased to 95.0±1.03%, 90.0±2.01% and 84.0±1.04% respectively after 90 days of exposure (Figure 1).

The body weight of control prawns was recorded as 25.0 ±1.75 g at the end of the experiment. TBT treatment found to decrease the body weight of prawns compared to control. At10ng/L of TBT treatment, the body weight of prawns was recorded as 23.0 ± 1.21 g. Besides, the body weights remarkably reduced to 17.0±0.72 g and 16.3 ±0.43 g at 100 ng/l and 1000 ng/l TBT, respectively at the end of experiment (Figure 1). Statistical analysis indicated that the changes in survival rate and body weight of TBT treated groups differed significantly to that of control group (P < 0.05).

### Assessment of reproductive activity

**Figure 2:**
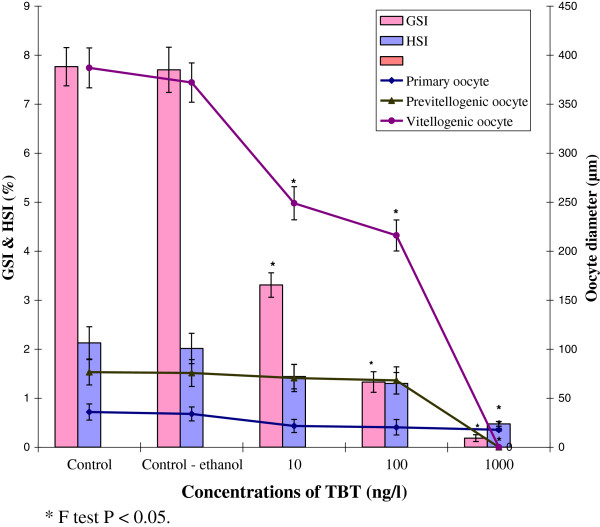
Impact of TBT on the GSI, HSI and oocyte growth in M. rosenbergii. * F test P < 0.05.

TBT had significantly reduced the GSI and HSI values in treated prawns. In control, the GSI and HSI values were recorded as 7.8 ±0.39% and 2.1 ±0.33% respectively. Whereas, the GSI and HSI values were steadily declined as the concentration of TBT increases. At higher concentration of 1000 ng/l TBT treated group, the GSI and HSI values were decreased drastically to 0.2±0.07% and 0.5 ±0.05% respectively after 90 days exposure (Figure 2).

### Oocyte growth

Oocyte growth has been affected by the treatment of TBT as evidenced by the decrease of oocyte diameter. In control prawn, the oocyte diameter measured to be 35.9±8.20 μm of primary oocytes, 76.5±12.90 μm of previtellogenic oocyte and 387.0 ± 20.4 μm of vitellogenic oocytes. In contrast, 10 ng/L of TBT exposure revealed 21.7±6.70 μm of primary oocyte, 70.5±13.8 μm of previtellogenic oocyte and 248.9±16.90 μm of vitellogenic oocyte. At 100 ng/L TBT treatment, the size of oocytes decreased to 20.3±7.90 μm, 68.1±13.90 μm and 216.0 ±15.80 μm of primary, previtellogenic and vitellogenic stages respectively. However at 1000 ng/L, TBT had retarded the oocyte development completely. Overall, the oocyte diameter decreased with an increase of TBT concentration (Figure 2). The changes in GSI, HSI values and oocyte diameter in treated groups were statistically significant from that of control groups (P < 0.05).

### Morphological variation of reproductive tissues

**Figure 3:**
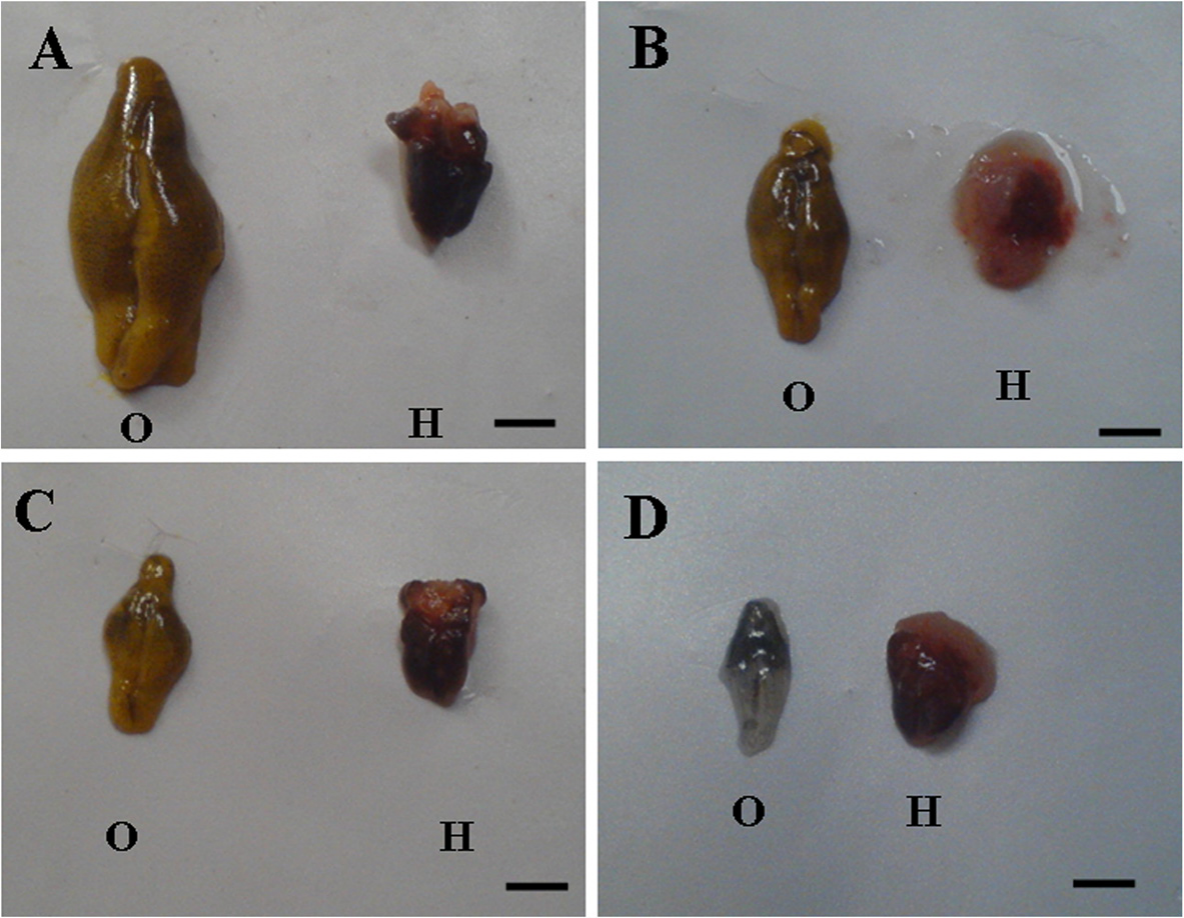
**(A) Prawn (control) showing fully mature vitellogenic stage ovary (O) and hepatopancreas (H). B**, **C**, **D** showing variation in the gross morphology of ovary and hepatopancreas in TBT treated prawns. (**B**) Note the reduction in the ovarian development proceeded up to early vitellogenic stage at 10 ng/l, (**C**) Becomes pre vitellogenic stage ovary at 100 ng/l and (**C**) No ovarian development at 1000 ng/l. Note the decrease in size of ovary and hepatopancreas in TBT treated prawns compared to control **(n = 3)**. Bar: 50 mm.

Morphological alterations have occurred in both hepatopancreas and ovary of TBT exposed prawns. Control prawns showed fully mature ovary representing late vitellogenic stage. At 10 ng/L TBT, the ovarian development proceeded up to early vitellogenic stage. In the intermediate concentration of 100 ng/L TBT, ovarian development was seen up to pre vitellogenic stage and thereafter the development was arrested. However, at higher concentration of 1000 ng/l TBT, the ovarian development was completely ceased and remained at spent stage. Moreover, hepatopancreas structure also distorted in all TBT treated groups. Gross morphology of both ovary and hepatopancreas also decreased apparently in TBT treated prawns compared to control (Figures 3A-D).

### Cellular level changes in different reproductive tissues

**Figure 4:**
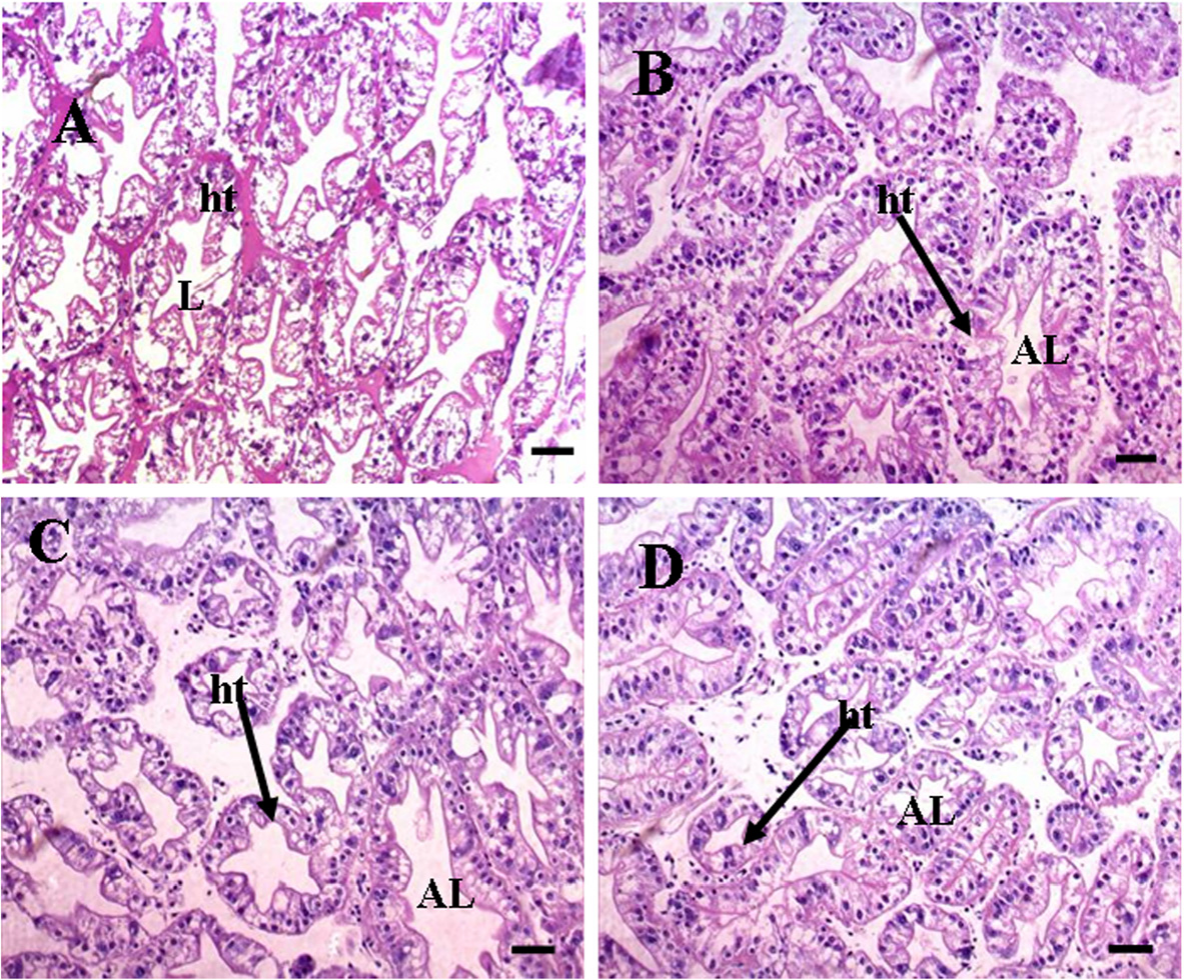
**(A) Section through hepatopancreas (control) showing the normal architecture of hepatopancreatic tubule (ht), lumen (L).** (**B**) At 10 ng/l TBT exposure, hepatopancreas showing reduction in the size of the hepatopancreatic tubule (↑ ht) and abnormal lumen (AL) (**C**) hepatopancreas showing reduction in the size of the hepatopancreatic tubule (↑ ht) and abnormal lumen (AL) at 100 ng/l TBT exposure, (**D**) At 1000 ng/l TBT exposure, hepatopancreas showing the reduction in size of the hepatopancreatic tubule (↑ ht), abnormal lumen (AL) and the disassociated epithelial cells from the hepatopancreatic tubules. Note the variation in size and arrangement of hepatopancreatic tubules in control and TBT treated groups **(n = 3)**. Bar: 50 μm.

Hepatopancreas of control prawn showed normal architecture of hepatopancreatic tubule, lumen and basement membrane thickness. However, on exposure to TBT (10 ng/l), the hepatopancreas exhibited swelling of hepatopancreatic tubule and abnormal lumen. Hepatopancreas of prawn treated with 100 ng/l TBT showed decrease in basement membrane thickness and disruption of hepatopancreatic tubules. At 1000 ng/l TBT, hepatopancreatic tubule size had reduced unusually and was seen with abnormal lumen. Besides disassociation of epithelial cells from the hepatopancreatic tubules was also observed (Figures 4A-D).

**Figure 5:**
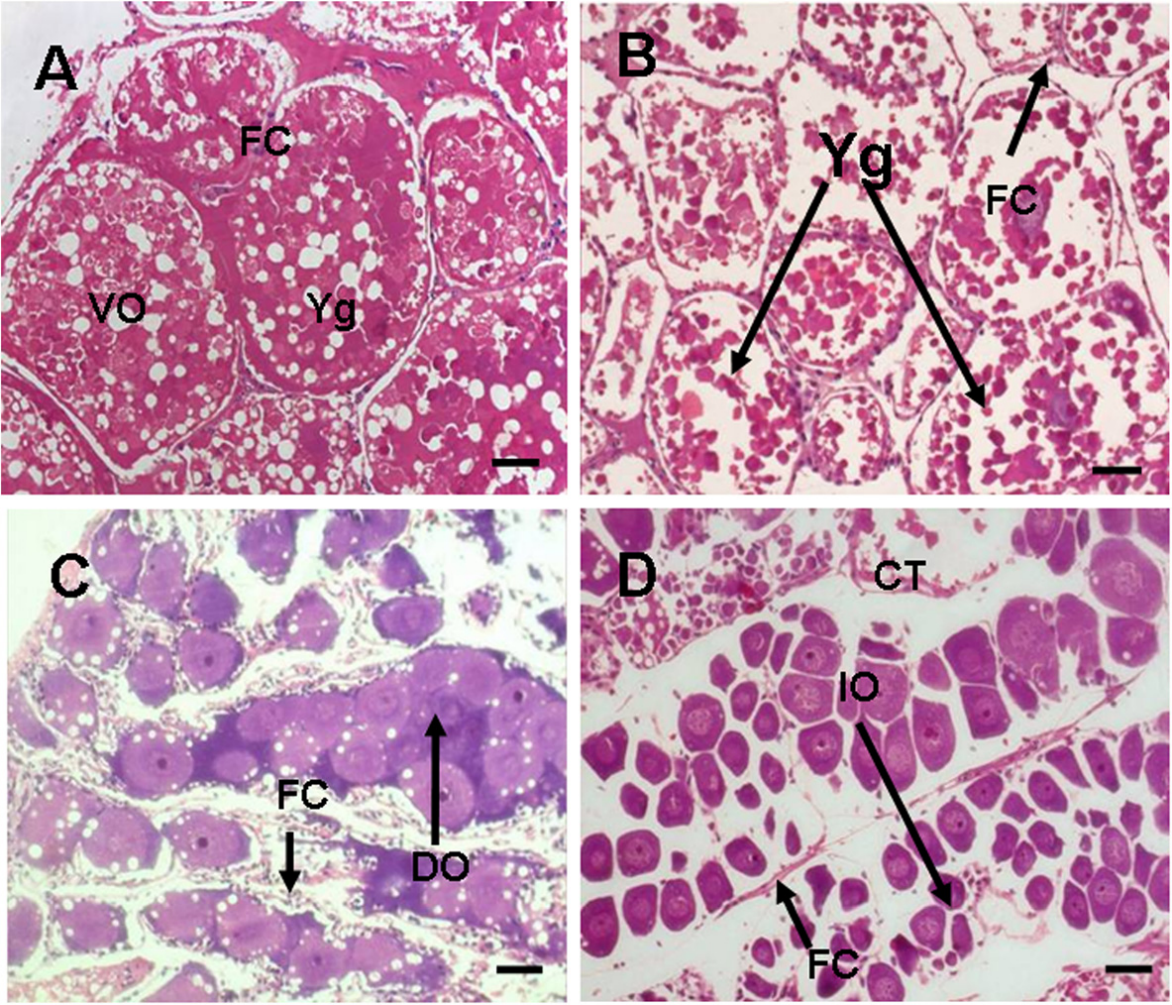
**(A) Section through ovary (control) showing vitellogenic oocytes (VO) with yolk globules (Yg) and encircled by a row of follicle cells (FC).** (**B**) At 10 ng/l TBT exposure, ovary show reduction in the yolk globules (↑Yg) and disruption of the follicle cells (↑ FC). (**C**) Ovary showing the fusion of developing oocytes (↑ DO) and disassociation of the follicle cells (↑ FC) at 100 ng/l TBT exposure, (**D**) At 1000 ng/l TBT exposure, ovary showing the degradation of connective tissues (CT), loss of follicle cells (↑ FC) and abundance of immature oocytes (↑ IO). Note the reduction in the number of developing oocytes in the treated ovary compared to control **(n = 3)**. Bar: 50 μm.

On the other hand, control prawn showed normal development of ovary with vitellogenic oocytes containing distinct ooplasm filled with yolk globules. The oocytes were enveloped by a row of characteristic follicle cells with prominent nucleus and nucleolus. At 10 ng/L TBT, the ovary showed previtellogenic oocytes and reduction in the yolk globules as well as disruption of follicle cells. At 100 ng/L TBT, ovary showed marked variation in the cellular architecture of oocytes such as fusion of developing oocytes and disassociation of follicle cells. At 1000 ng/L TBT, the ovary showed immature oocytes and reduction in size of yolk material (Figures 5A-D).

### Identification of vitellin in ovary

**Figure 6:**
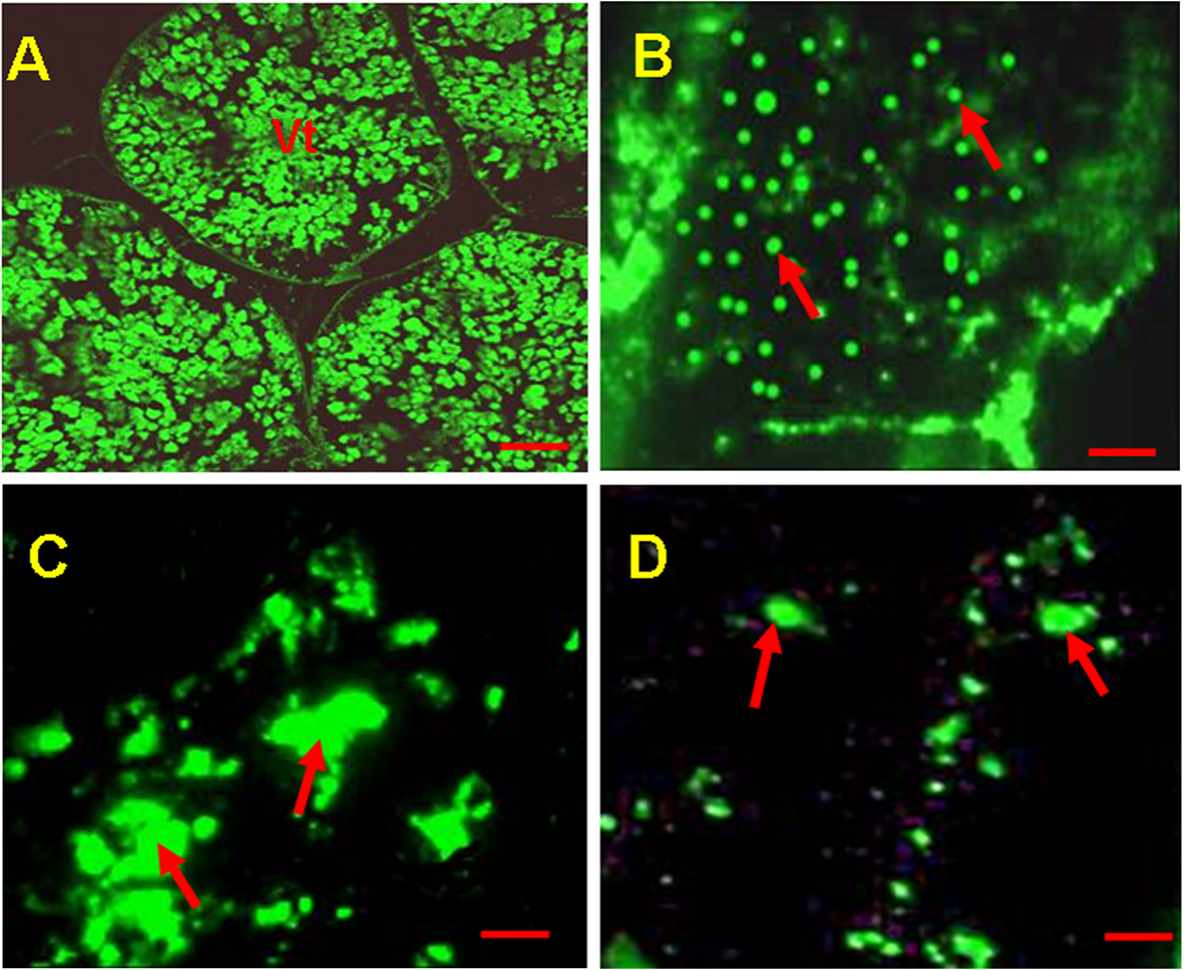
**Immunofluorescence expression of vitellin in ovary (n = 3).** (**A**). Control prawn ovary showed maximum Immunostaining expression of vitellin (Vt) (**B**). Moderate expression of vitellin (↑) at 10 ng/l exposure (**C**). Less expression of vitellin (↑) at 100 ng/l exposure (**D**). At 1000 ng/l, note the very low expression of vitellin (↑). Bar: 50 μm.

The results of immunofluorescence study with specific antivitellin (primary antibody) and FITC conjugation (secondary antibody) clearly indicated the high amount of vitellin content as a measure of increased immunostaining with anti vitellin and FITC in control. Besides moderate to less immunostaining in the ovary of TBT (10 ng/L) treated prawn indicated less vitellin content. Interestingly, at 100 ng/L of TBT, ovary showed fusion and reduction of vitellin content as evident with immunostaining. At higher concentration of TBT (1000 ng/l), very low intensity of vitellin content was observed in the ovary (Figures 6A-D).

### Biochemical variations in different reproductive tissues

In the 90 days of TBT exposure, marked reduction in protein content in all reproductive tissues were recorded in TBT treatment from 10 ng/l to 1000 ng/l. Protein content had significantly decreased to 19-fold in hepatopancreas, 22-fold in ovary and 1.5-fold in hemolymph at 1000 ng/L concentration of TBT compared to control. Statistical analysis revealed that the variation of protein content in TBT exposed prawns differed significantly(P < 0.05) than that of control group (Table 1).

**Table 1:** **Impact of TBT on the biochemical constituents (n = 5) in different reproductive tissues of *M. rosenbergii***

**Biochemical constituents in test tissues**	**Control groups**		**TBT (ng/l) treated experimental groups**		
	**Control**	**Control ethanol**	**10**	**100**	**1000**
**Protein content**					
Hepatopancreas	48.5±1.01	47.1±1.95	42.0±1.02	34.0±2.81^*^	26.0±2.95^*^
Ovary	98.6±1.17	97.3±1.15	65.7±4.21	37.7±2.15^*^	4.4±0.25^*^
Hemolymph	170.5±4.50	169.3±2.90	165.1±3.00	142.7±2.10	110.1±1.10^*^
**Lipid content**					
Hepatopancreas	15.7±1.27	15.1±2.01	13.3±3.70	11.7±0.51^*^	10.8±0.45^*^
Ovary	56.5±0.21	56.1±0.97	45.9±2.15	35.9±1.28^*^	3.2±0.15^*^
**Glycogen content**					
Hepatopancreas	25.8±0.83	25.5±0.47	23.1±0.17	21.8±0.42^*^	19.1±0.36^*^
Glucose content					
Hemolymph	25.8±0.37	25.3±0.58	28.1±0.38	29.6±0.25^*^	30.1±0.89

$\bar{X}\pm \mathrm{SD}$ of five observations.

* F-test P < 0.05.

Total lipid content in the TBT treated prawn also decreased in tested tissues. Lipid content in hepatopancreas and ovary had remarkably decreased in 1000 ng/L TBT treated group. A decrease of 1.5-fold in hepatopancreas and 17.5-fold in ovary was registered after 90-days of exposure to 1000 ng/l over control (Table 1). Variations in the lipid content in TBT treated groups differd significantly from that of control groups (P < 0.05).

The prawn exposed to TBT showed decrease in the glycogen content compared to control. At the end of experiment, glycogen content had notably decreased to 1.3-fold in hepatopancreas at higher concentration of 1000 ng/l TBT than control (Table 1). Statistical analysis inferred that variation of glycogen content in TBT treated groups differd significantly from that of control group (P < 0.05).

Interestingly, the glucose content increased in hemolymph of all TBT treated groups compared to control. Glucose content increased to 0.9-fold in 1000 ng/L TBT treated group (Table 1). Statistical analysis inferred that glucose content significantly (P < 0.05) increased in TBT treated groups compared to control group.

### Quantification of vitellogenin and vitellin content

**Figure 7:**
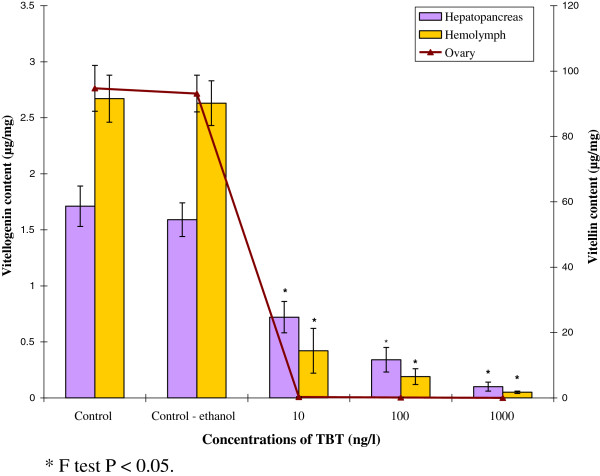
Effect of TBT on the vitellogenin and vitellin content in M. rosenbergii. * F test P < 0.05.

The results clearly indicated that vitellogenin and vitellin content decreased significantly from the exposure of TBT, compared to control. In control prawn, the vitellogenin content in hepatopancreas and hemolymph was recorded as 0.7±0.15 μg/g and 2.5±0.36 μg/mL respectively. Interestingly, at higher concentration of TBT (1000 ng/l), vitellogenin content reduced drastically in both hepatopancreas (0.3±0.14 μg/g) and hemolymph (0.50±0.32 μg/ml) after 90 days of exposure. On the other hand, vitellin content in the treated prawns remarkably decreased from 18.7±4.12 μg/g at 10 ng/l to 0.2±0.11 μg/g at 1000 ng/l TBT (Figure 7). The variation of vitellogenin and vitellin content in TBT treated groups differd significantly from that of control group (P < 0.05).

### Quantification of sex hormones

#### 17β-estradiol level in different reproductive tissues

**Figure 8:**
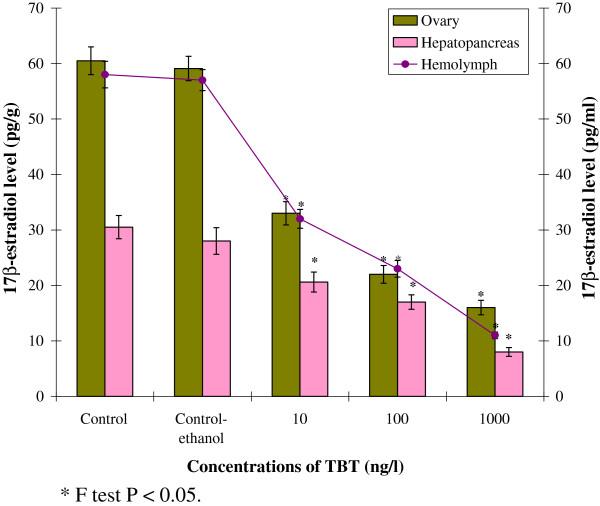
Effect of TBT on the 17β-estradiol in different reproductive tissues of M. rosenbergii. * F test P < 0.05.

In TBT treated prawns, 17β-estradiol level decreased in all reproductive tissues compared to control (Figure 8). In control prawn, 17β-estradiol levels in ovary, hemolymph and hepatopancreas was recorded as 60.5±2.50 pg/g, 58.0±2.10 pg/ml and 30.5±2.40 pg/g respectively. On exposure to TBT (10 ng/l), 17β-estradiol level reduced to 33.0±2.10 pg/g in ovary, 32.0±1.80 pg/ml in hemolymph and 20.6±1.70 pg/g in hepatopancreas. At 100 ng/L TBT, 17β-estradiol level in ovary, hemolymph and hepatopancreas amounted to 22.0±1.60 pg/g, 23.0±1.30 pg/ml and 17.0±1.50 pg/g, respectively. However, at higher concentration of TBT (1000 ng/l), 17β-estradiol level decreased significantly in ovary (16.0±1.30 pg/g), hemolymph (11.0±0.80 pg/ml) and hepatopancreas (8.0±0.60 pg/g). The variation of 17β-estradiol level in control and treated groups at higher concentration was significant (P<0.05).

### Testosterone level in ovary

**Figure 9:**
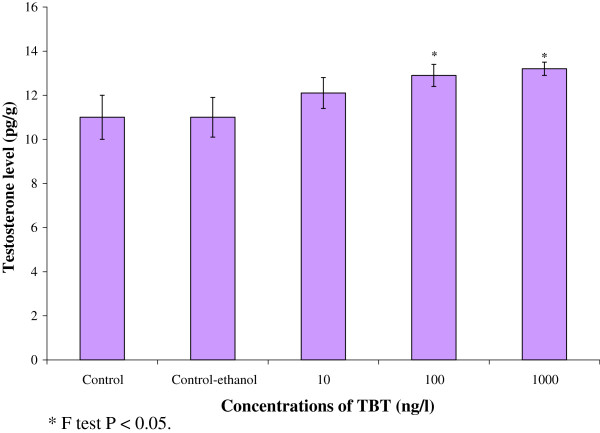
Effect of TBT on the testosterone in ovary of M. rosenbergii. * F test P < 0.05.

The level of testosterone gradually increased in the ovary of TBT treated groups (Figure 9). The testosterone level in the ovary of control prawns was recorded as 11.3±1.60 pg/g. The testosterone level showed a marginal increase of 12.1±0.70 pg/g and 12.9±0.50 pg/g at 10 ng/L and 100 ng/L respectively. At higher concentration of TBT (1000 ng/l), testosterone level increased to 13.2±0.30 pg/g in the ovary. The testosterone levels significantly increased in treated groups compared to control (P<0.05).

## Discussion

Our results clearly demonstrated that TBT had considerably reduced the survival rate as well as growth in terms of body weight in all TBT treated prawns compared to control. Similarly, Lignot et al. [18] reported that TBT had significantly reduced the survival rate in *Penaeus japonicus*. The weight of prawn decreased to 65.2-fold at higher concentration in TBT treated group. The developmental rate slowdown in all TBT treated groups and even lower concentrations had effect on growth in *M. rosenbergii*. Likewise, Laughlin et al. [19] also described the effect of TBT on growth in mud crab, *Rhithropanopeus harrisii*. Lobster larvae (*Homarus americanus*) exhibited decreased growth and increased mortality at 1 μg/L [20]. Similarly, in our previous study also reported that TBT had substantially reduced the growth rate in *M. rosenbergii*[11].

The present study clearly demonstrated that TBT had impaired the ovarian development as evidenced by the level of GSI, HSI indices and oocyte diameter in *M. rosenbergii*. Likewise, Zhang et al. [21] suggested that TBT can affect the GSI and HSI in female *Sebastiscus marmoratus*. The results revealed the dose dependent toxicity of TBT on the reproductive performance of *M. rosenbergii.* The higher concentration of TBT (1000 ng/l) had entirely arrested the ovarian development and led to spent stage in *M. rosenbergii*. Similar observation has been reported in freshwater prawn *M. rosenbergii* when exposed to cadmium [22]. Rodriguez et al. [23] also documented the impairment of ovarian development after exposure to cadmium in fiddler crab, *Uca pugilator*.

In crustaceans, the hepatopancreas are considered to function as a storage organ of organic nutrients in the form of protein, lipid and carbohydrate, which are mobilized during the reproductive cycle, in order to meet the specific requirements of maturing gonads [24]. After exposure to TBT, the hepatopancreas exhibited rumpling of basement membrane, abnormal lumen, disorganization of epithelial cells, disruption in the hepatopancreatic tubule and occurrence of more number of vacuoles. These cellular and structural damages in hepatopancreas of *M. rosenbergii* significantly affect the absorption, secretion, digestive functions and the production of precursor material for major yolk protein; vitellogenin synthesis. Likewise, Sreeram and Menon [25] reported that the function of hepatopancreas of *Metapenaeus dobsoni* affected by the exposure of petroleum hydrocarbons (PHC). Klobucar et al. [26] also found swelling and damage of cell membranes in digestive gland of snail *Planorbis corneus* exposed to polychlorinated biphenyls (PCB).

From the present study, it was obvious that, TBT had substantially decreased and retarded the sexual maturation in *M. rosenbergii*. It also affected the ovarian development, as indicated by the reduction in oocyte diameter, yolk globules, fusion of developing oocytes and disruption of follicle cells. Sex steroid hormones are synthesized by follicle cells in the ovaries of female fish [27]. These follicle cells are sensitive to environmental stressors. It has been demonstrated that some natural and xenobiotic chemicals induce apoptosis in follicle cells [28]. The results of the current study clearly revealed that the follicle cells had remarkably affected and this may cause impairment in the steroid synthesis in experimental prawns. In the ovary, 17β-estradiol is catalyzed by the steroid synthesizing enzymes, in particular the P450 aromatase. In teleosts, change in the activity and expression of P450 aromatase have been shown to drive changes in the ovarian production of 17β-estradiol during the reproductive cycle [29].

The above hypothesis was consistent to the present study as the inhibition of gonadal maturation associated with increased levels of testosterone and decreased levels of 17β-estradiol in the ovary of prawns exposed to TBT, which should be accompanied with inhibition of P450 aromatase activity. Thus the changes of sex hormone levels would finally influence the ovarian development of prawn. In recent years, there is an upsurge in the interest of toxicity on the endocrine disruption in crustaceans which ultimately affect the process of gametogenesis. Accordingly, Zhang et al. [21] reported that environmentally realistic concentrations of TBT had an adverse effect on ovarian development in cuvier *S. marmuratus*. Here, ovary showed several spherical structures formed by the fragmentation of nucleus lying randomly in the chromatin network, yolk vesicles at the periphery, vacuolization in the nucleus, stromal hemorrhage and damage of germinal epithelium. Histo-anatomical abnormalities in ovaries may be caused by xenobiotics [30].

The alteration in basic biochemical metabolism ultimately posing a detrimental effect on growth and reproductive activity of *M. rosenbergii* exposed to TBT. Major biochemical constituents such as protein and lipid content also decreased in all the treated groups. The depletion of protein content suggests the increase in proteolysis and possible utilization of the product of their degradation for metabolic purpose. Products of proteolysis may be mobilized into trichloroacidic acid cycle through amino acid metabolism to cope up with the excess demand of energy during toxic stress. The fall in protein level during pollutant exposure may be due to increased catabolism and decreased anabolism of proteins. Similarly, depletion of protein content in different tissues of *Macrobrachium kistnensis* exposed to TBTCL was reported by Kharat et al. [1]. The decrease in protein and lipid content was reported in freshwater prawn *M. kistnensis* exposed to organotin pesticides [31]. The total lipid content also decreased because of the utilization of fatty acid deposits instead of glucose for energy purpose under TBT toxicity. Accordingly, West et al. [32] reported the decrease of total lipid content in hepatopancreas with increased activity of lipase, the enzyme is responsible for the breakdown of the lipid into free fatty acids and glycerol. Since lipids form a rich energy reserve whose caloric value is reported to be twice that of an equivalent weight of carbohydrate or proteins, the mobilization of lipid reserve testifies the imposition of high energy demands. Therefore, the amount of total lipid found to be lower in tissues of prawns exposed to TBT.

In the present study, a noticeable reduction of glycogen content in the hepatopancreas resulted the elevation of glucose level in hemolymph exposed to TBT. The decrease in glycogen content may be due to enhanced breakdown of glycogen to glucose through glycogenolysis under toxic stress of TBT in *M. rosenbergii*. Depletion in glycogen level might be due to its rapid utilization to meet the energy demands under toxic stress and to supply energy demand in the form of glucose which undergoes breakdown to produce energy rich ATP. Similarly, depletion of glycogen content in hepatopancreas of *M. kistnensis* exposed to TBTCL was reported by Kharat et al. [1]. Holwerda and Herwig [33] also reported that the dibutyltinchlorine exposed clam *Anodonta anatine,* showed notable decrease in carbohydrate content. In crustaceans, elevation of hemolymph glucose was also observed *In vivo* when they were subjected to pollutants [34]. Chin-Chyuan Chang et al. [35] found that the depletion of glycogen stores should be accompanied by an increase in glucose content after exposure of *M. rosenbergii* to trichlorfon. The glucose content increased with an increase of trichlorfon concentrations based on the dose-dependent manner.

Our results clearly demonstrated the impact of TBT on the vitellogenesis by reducing the vitellogenin (Vg) and vitellin (Vt) content in *M. rosenbergii*. Low concentration of Vg and Vt is an indication of malfunction of reproductive activity resulted in inhibition of reproductive performance in *M. rosenbergii*. Similarly, Vijayavel et al. [36] also found that the reduction in vitellogenin content influenced by naphthalene stress. Vitellogenin content can also act as a biomarker to study the abnormality in vitellogenesis. Low level of vitellogenin content is an indication of malfunction of reproductive endocrine system and inhibited the ovarian development [37].

Overall, the results clearly demonstrated the increased testosterone level and decreased 17β-estradiol levels in the ovary of prawns exposed to TBT, which should be associated with an inhibition with P450 aromatase activity. Thus the changes of sex hormone levels will influence the ovarian development of prawn. Zhang et al. [21] reported similar changes in ovarian development as well as sex hormonal changes in *S. marmoratus.*

## Conclusion

Thus, TBT had reduced the vitellogenin and vitellin content by retarding vitellogenesis. On the other hand, imbalance of sex hormones such as decrease in 17β-estradiol and increase in testosterone has led to endocrine imbalance and ultimately that would have impaired the oogenesis in the freshwater female prawn *M. rosenbergii*.

## Methods

### Collection and maintenance of prawn

Freshwater female prawns, *M. rosenbergii* were collected from the Aqua Nova hatchery in Kannathur near Chennai, South India. The collected prawns were brought to the laboratory in plastic cover with habitat water. They were introduced into plastic tanks with sufficient aeration. The water was changed daily and prawns were fed *ad libitum* with commercial pelletized feed. They were maintained in the laboratory for 2-3 weeks for acclimatization.

### Experimental design and TBT treatment

Five months old prawns (75 individuals with a weight of 16 ± 2g/individual) were selected and further divided in 5 groups (15 individuals or prawns/group).The first group served as control (without any treatment). As ethanol is a solvent used to prepare the TBT solutions, the second group served as positive control that received 2% ethanol treatment. Remaining three groups were exposed to TBT (10,100,1000 ng/l). Every day, the water was exchanged and the nominal concentrations of TBT were maintained in experimental tanks. For each treatment, triplicates were maintained and the experiment was conducted for a period of 90 days with the water temperature of 18 ± 2°C. Experimentation with invertebrates like prawn does not require any ethical approval.

### Assessment of reproductive activity

At the end of the experiment, the prawns were weighed, gonads removed and the weight of the gonads were recorded. The Gonado Somatic Index (GSI) and Hepato Somatic Index (HSI) were calculated following the procedure outlined by Zhang et al. [21].

### Measurement of oocyte development

Oocyte diameter was measured using an ocular micrometer calibrated with a stage micrometer fitted in a light microscope (Labex, India). For each prawn, the diameters of at least 30 oocytes were measured and mean oocyte diameter was calculated. The stage of oocyte development was characterized based on the maximum number of oocytes confined to a particular stage of development. Photomicrographs of various stages of oocyte development were taken using Leica 2500 microscope (Germany).

### Histology

Triplicate histological analyses were done by sacrificing three animals from each group. For this, the reproductive tissues such as hepatopancreas and ovary were dissected out carefully. The tissue samples were fixed in Bouin’s fixative for 24h and washed with distilled water. The samples were dehydrated with different graded alcohol series and processed by routine procedure. Sections of 6-8 μm thickness were taken and stained with haematoxyline and eosin. The stained sections were mounted using DPX and photomicrographs of varying magnifications were taken using Leica 2500 microscope.

### Immunofluorescence

For immunofluorescence study, ovaries (control and TBT exposed) were fixed in 4% paraformaldehyde in phosphate buffer saline (PBS) (pH7.0) at 4°C overnight. After washing with PBS (pH 7.0) three times, the samples were immersed in 30% saccharose-PBS buffer overnight at 4°C. They were then embedded in wax and sectioned at 6-7μm thickness using microtome (Leica). Then the sections were dehydrated in PBS for 30 min and incubated for 1h with 5% dry milk in PBS at room temperature to prevent non-specific binding of antibodies. The sections were then incubated over night at 4°C with the specific primary antibody (rabbit antibody) for vitellogenin (1:2000 dilution). The slides were washed with PBS, subsequently incubated for 1h with fluorescein iso thiocyanate (FITC) conjugated secondary antibody (anti-rabbit IgG, 1:100 dilution) in the dark and washed five times with PBS (10min each). Then the sections were stained by propidium iodide (PI) for 5min and washed four times (5min each). Finally, the sections were observed under Leica confocal fluorescence microscope.

### Biochemical analysis

#### Protein

After 90 days of treatment with TBT, prawns were dissected and test tissue samples (hepatopancreas, ovary and hemolymph) were taken and used for total protein content estimation by Coomassie Brilliant Blue G–250 method as described by Bradford [38].

#### Lipid

The total lipid content in hepatopancreas and ovary was analyzed using the Vanillin –Phosphoric acid method according to Folch et al. [39].

#### Glycogen

Glycogen content in the hepatopancreas was quantified following the method of Dezwann and Zandee [40].

#### Glucose

Glucose content in hemolymph was estimated following the procedure of Tietz [41].

### Isolation of vitellogenin and vitellin

Vitellogenin and vitellin were isolated from the hepatopancreas, hemolymph and ovaries of prawn *M. rosenbergii* following the method of Tsukimura et al. [42]. In brief, the reproductive tissues were homogenized in homogenization buffer (containing 0.1M NaCl, 0.05M Tris, 1mM ethylene diamine tetra acetic acid and 0.1% Tween 20 with 10mg/ml PMSF; pH 7.8) using an ice cold glass homogenizer. The homogenate was centrifuged at 4000 × g for 5 min at 4°C. The resultant supernatant was again centrifuged at 20,000 × g for 20 min at 4°C. To the supernatant, saturated ammonium sulphate was added to produce 25% SAS solution. After incubation for 1h at 4°C, the solution was centrifuged at 20,000 × g for 10 min at 4°C. The supernatant was collected and saturated ammonium sulphate was added to produce 40%, 50% and 60% saturated ammonium sulphate solution sequentially. The pellets of 60% saturated ammonium sulphate solution was suspended in appropriate volume of homogenization buffer and dialyzed thrice at 4°C for 12h each against homogenization buffer. Further, the isolated vitellogenin and vitellin were purified by following the scheme of Zagalsky et al. [43]. Then the purified vitellogenin and vitellin were stored at -20°C for further analysis.

### Enzyme linked immunosorbent assay

Hundred milligrams of hepatopancreas, ovary and hemolymph samples were taken individually from control and TBT treated groups. Tissues were individually homogenized with phosphate buffer and centrifuged at 13,000 × g for 10min at 10°C, to remove cellular debris. The supernatant was collected in separate vials and stored at -20°C until assay. Microtitre plates were filled with 100μl (six replicates) of different samples separately, diluted with coating buffer and incubated over night at 4°C. After three washings with buffer, the wells were blocked with 200μl of blocking buffer and incubated at 37°C for 1h. Washing was followed by the addition of 100μl of primary antibody (anti Vg at 1: 2000), for 3h at 37°C. The primary antibody was priorly raised in rabbit using the purified Vg from *M. rosenbergii*. After three times washing, the wells were coated with 100μl secondary-antibody enzyme conjugated (anti rabbit IgG-Alkaline phosphatase) at 1: 500 dilutions for 1h at 37°C. Incubation was terminated by washing and wells were filled with 100μl of substrate solution (1mg pNPP - paranitrophenyl phosphate/ml of substrate buffer). The reaction was stopped with the stop buffer after the required colour development was attained. Concentrations of Vg standard was ranged from 0.1 - 100μg/ml. Absorbance at 405nm was measured in an automated ELISA plate reader (Titertek Multiscan Plus, MK II, Denmark).

### Hormonal assay

#### Radioimmunoassay

The steroid extract of ovary, hemolymph and hepatopancreas was estimated for the level of free immunoreactive 17β**-**estradiol and testosterone using radioimmunoassay (RIA) according to the protocol of Oreczyk et al. [44]. The steroid extracts (six replicates/each sample) were reconstituted separately in 100 μl of gelatin phosphate buffer solution (GPBS) (sodium phosphate buffer 0.1M, pH 7.2, containing 0.15 M NaCl and 0.1% gelatin) in RIA tubes. Appropriately diluted antiserum to 17β**-**estradiol and testosterone (New England Nuclear Corp., Boston, MA) and 0.1 ml of [^3^H]-steroids without antiserum (to determine non-specific binding) were included in every assay. At the end of incubation, bound and free steroids were separated by adding 0.3 ml of dextran coated charcoal (0.1% dextran T70 and 1% charcoal in PSB) and each tube was centrifuged at 3000 × g for 20 min at 4°C. The supernatant was poured carefully without disturbing the charcoal pellet into the vials containing 5 ml of scintillation fluid (0.5% PPO, 0.04% POPOP and 25% methanol I toluene). The vials were shaken at room temperature to extract steroids into aqueous phase and steroid levels were estimated using a liquid scintillation counter (Beckman, USA).

### Statistical analysis

Data obtained on the biochemical and hormonal analyses of both control and treated prawns were subjected to statistical analyses, such as one way analysis of variance (ANOVA) and F-test using SPSS 7.5 to determine whether the variations between the groups were significant.

## Abbreviations

TBT: Tributyltin; EDC: Endocrine disrupting compound; GSI: Gonado somatic index; HSI: Hepato somatic index; PHC: Petroleum hydrocarbons; PCB: Polychlorinated biphenyls; FITC: Fluorescein Iso ThioCyanate; PI: Propidium iodide; RIA: Radioimmunoassay; GPBS: Gelatin phosphate buffer solution.

## Competing interests

The authors declare that they have no competing interests.

## Authors’ contributions

PI assisted in performing immunological assays done in the present study. LAV helped to carryout the hormonal assay part of this work. NM and MK supervised and helped in drafting MS with their critical interpretations. All authors read and approved the final MS for publication.
